# Biocompatibility, bioactivity, porosity, and sealer/dentin interface of bioceramic ready-to-use sealers using a dentin-tube model

**DOI:** 10.1038/s41598-024-66616-7

**Published:** 2024-07-22

**Authors:** Rafaela Nanami Handa Inada, Evelin Carine Alves Silva, Camila Soares Lopes, Marcela Borsatto Queiroz, Fernanda Ferrari Esteves Torres, Guilherme Ferreira da Silva, Paulo Sérgio Cerri, Juliane Maria Guerreiro–Tanomaru, Mário Tanomaru-Filho

**Affiliations:** 1 School of Dentistry, Department of Restorative Dentistry, São Paulo State University (UNESP), Araraquara, 14801‑903 Brazil; 2 Department of Dentistry, Centro Universitário Sagrado Coracao (UNISAGRADO), Bauru, 17011-160 Brazil; 3 Laboratory of Histology and Embryology, Department of Morphology, Genetics, Orthodontics and Pediatric Dentistry, School of Dentistry, São Paulo State University (UNESP), Araraquara, 14801‑903 Brazil

**Keywords:** Dental materials, Endodontics

## Abstract

This study evaluated the biocompatibility, bioactivity, porosity, and sealer/dentin interface of Sealer Plus BC (SP), Bio-C Sealer (BIOC), TotalFill BC Sealer (TF), and AH Plus (AHP). Dentin tubes filled with the sealers and empty tubes (control group) were implanted in the subcutaneous tissue of rats for different periods (n = 6 per group/period). Number of inflammatory cells (ICs), capsule thickness, von Kossa reaction, interleukin-6 (IL-6) and osteocalcin (OCN) were evaluated. Porosity and voids in the interface dentin/sealers were assessed by micro-computed tomography. The data were submitted to ANOVA/Tukey’s tests (α = 0.05). Greater capsule thickness, ICs and IL-6 immunolabeling cells were observed in AHP. No significant difference in thickness of capsule, ICs, and IL-6- immunolabeling cells was detected between SP and TF, in all periods, and after 30 and 60 days between all groups. At 60 days all groups had reduction in capsule thickness, ICs and IL-6 immunolabeling cells. Von Kossa-positive and birefringent structures were observed in the capsules around the sealers. BIOC, SP, and TF exhibited OCN-immunolabeling cells. All sealers had porosity values below 5%, besides low and similar interface voids. BIOC, SP and TF are biocompatible, bioactive, and have low porosity and voids. The dentin-tube model used is an alternative for evaluating bioceramic materials.

## Introduction

Adequate physicochemical and biological properties of bioceramic root canal sealing materials are essential for the success of endodontic treatment^1^. Ready-to-use endodontic sealers based on calcium silicates have been developed, such as Bio-C Sealer (BIOC; Angelus Indústria de Produtos Odontológicos, Londrina, PR, Brazil), Sealer Plus BC (SP; MK Life, Porto Alegre, PR, Brazil), and TotalFill BC Sealer (TF; FKG Dentaire AS, La Chaux-de-Fonds, CH, Switzerland). These sealers have been demonstrated biological properties, such as biocompatibility, bioactivity, and cytocompatibility^2–5^, besides adequate physicochemical properties, except for a high solubility^6,7^. On the other hand, AH Plus (AHP; Dentsply DeTrey GmbH, Konstanz, BW, Germany) is an epoxy resin-based sealer considered as gold standard^8^ due to their excellent physicochemical properties, including low solubility^9^.

Polyethylene tubes implantation in subcutaneous of rats is indicated by the International Organization for Standardization (ISO-10993-6)^10^ to evaluate the biocompatibility and bioactivity of calcium silicate-based endodontic materials^3,4,11^. However, polyethylene tubes have limitations for studies with calcium silicate materials, as they do not allow dentin/biomaterial interactions^12,13^.

Dentin tubes can be used to evaluate the properties of endodontic materials due to their moisture, which is necessary for the setting of sealers based on calcium silicate^14^. Furthermore, the dentin tube is well tolerated in the subcutaneous tissue of rats^15^. It is also important to consider that materials based on calcium silicate are potentially bioactive when placed in direct contact with dentin and tissue fluids^16,17^ leading to the formation of apatite^18^, and an interfacial layer with tag-like structures between bioceramic material and dentin^13^. Although this model has proven to be appropriate for evaluating repair cements based on calcium silicates^19^, until now, there are no studies evaluating bioceramic sealers using the dentin tube model in the subcutaneous of rats.

In addition to biological characteristics, physicochemical properties of root canal sealers must be evaluated^20^. Micro-computed tomography (micro-CT) is a highly accurate, non-destructive tool that has been used to measure the porosity inside the endodontic sealers^21,22^ and to evaluate the percentage of gaps and voids in the interface between the dentin wall and endodontic sealers^19,23^. However, there are still no studies evaluating the volumetric properties of root canal sealers when implanted in the tissular fluid of subcutaneous of rats.

The analysis of the biological and physicochemical properties using methodologies similar to the clinical condition such as dentin tubes implantation in addition to tests using micro-CT are important methodologies that can be applied in studies of bioceramic materials^19^. Therefore, the aim of the present study was to evaluate the biocompatibility, bioactive potential, porosity, and interface dentin-sealer of BIOC, SP and TF compared with AHP, using the dentin tube model in the subcutaneous tissue of rats. The null hypothesis is that the sealers have no difference regarding to biological and physicochemical properties (Table 1).

**Table 1:** Bioceramic sealers, chemical composition, manufacturer, and proportions.

Sealer	Composition	Manufacturer	Proportion
Bio C-Sealer (BIOC)	Calcium silicates, calcium aluminate, calcium oxide, zirconium oxide, iron oxide, silicon dioxide, and dispersing agent	Angelus, Londrina, Brazil	Ready-to-use
Sealer Plus BC (SP)	Calcium disilicate, nanoparticulate calcium trisilicate, zirconium oxide	MK Life, Porto Alegre, Brazil	Ready-to-use
TotalFill BC Sealer (TF)	Zirconium oxide, calcium silicates, calcium, monobasic phosphate, calcium hydroxide, filling, and thickening agents	FKG Dentaire SA, La Chaux-de-Fonds, Switzerland	Ready-to-use
AH Plus (AHP)	Paste A: epoxy bisphenol-A resin and epoxy bisphenol-F, calcium tungstate (WO4Ca2), zirconium oxide (ZrO2), silica, iron oxide. Paste B: dibenzyl amine, aminoadamantane, WO4Ca2, ZrO2, silica, silicone	Dentsply DeTrey GmbH, Konstanz, Germany	1 g: 1 g

## Results

Descriptive statistics of the biological properties as well as porosity and interface analysis data were performed using mean and standard deviation values.

### Morphological and morphometrical findings

**Figure 1:**
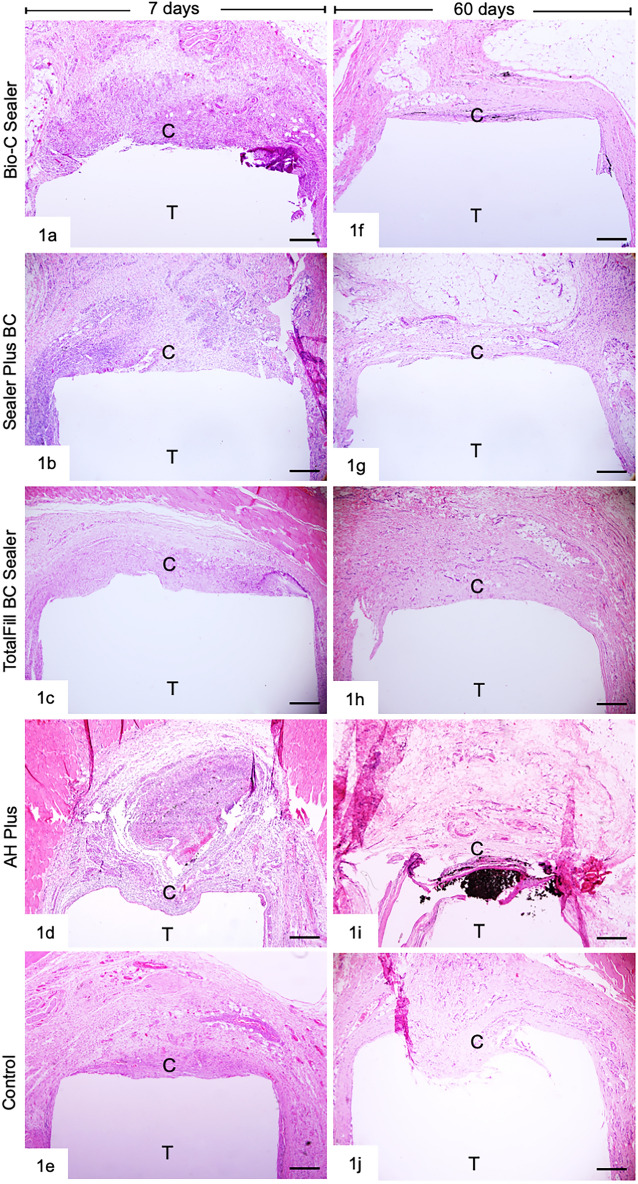
Photomicrographs of sections showing portions of capsules (C) adjacent to the opening of the dentin tubes (T). Figures 1a–j: an overview of the capsules (C) after 7 (Fig. 1a–e) and 60 (Fig. 1–j) days to the implants. HE. Bars = 50 µm.

The sections of capsule thickness of the sealers in different periods are presented in Fig. 1. At 7 days, the dentin tubes were surrounded by thick capsules exhibiting a high density of cells. Note that in the AHP specimens, an intense inflammatory infiltrate was present. However, in all groups, the inflammatory infiltrate was located next to the opening of the dentin tubes and adjacent tissues (loose connective tissue and muscle tissue) were with standard features (Fig. 1a–e). In addition, an evident reduction in the thickness of the capsules was observed in groups after 60 days (Fig. 1f–j).

According to Table 2, the thickness of capsules of AHP was significantly greater than in other groups in all periods (*P* < 0.05). At 7 and 15 days, the capsules around BIOC specimens were significantly thicker than in SP and TF specimens (*P* < 0.05). Moreover, no significant difference was found among SP, TF and CG specimens at 15 days (*P* > 0.05). From the 30th BIOC, SP, TF, and CG specimens exhibited thin capsules, and no significant difference was detected among these groups (*P* > 0.05). The capsules of the SP, TF, and AHP groups reduced over time while the BIOC reduced at 15 days when compared to 7 days, and CG reduced at 30 days (*P* < 0.05). All the groups presented the lowest values of capsule thickness at 60 days.

**Table 2:** Mean and standard deviation of capsule thickness (µm), number of inflammatory cells (ICs), of interleukin-6- immunolabelled cells (IL-6) and of osteocalcin-immunolabelled cells (OCN) per mm^2^ in the capsules adjacent to the different materials and control group after 7, 15, 30, and 60 days.

	BIOC	SP	TF	AHP	CG
7 days					
Capsule thickness	501 ± 35^b,1^	395 ± 43^c,1^	386 ± 49^c,1^	625 ± 69ª^,1^	299 ± 66^d,1^
ICs	887 ± 95^b,1^	472 ± 64^c,1^	482 ± 21^c,1^	1171 ± 90ª^,1^	374 ± 99^d,1^
IL-6	300 ± 22^b,1^	257 ± 8^c,1^	228 ± 14^c,1^	380 ± 26ª^,1^	144 ± 12^d,1^
OCN	39 ± 6ª^,1^	17 ± 6^c,1^	28 ± 6^b,1^	–	–
15 days					
Capsule thickness	385 ± 30^b,2^	291 ± 44^c,2^	305 ± 35^c,2^	469 ± 21^a,2^	276 ± 34^c,1^
ICs	571 ± 50^b,2^	414 ± 64^c,1^	415 ± 14^c,1^	686 ± 33ª^,2^	316 ± 41^d,1^
IL-6	219 ± 12^b,2^	152 ± 21^c,2^	150 ± 11^c,2^	270 ± 17ª^,2^	117 ± 13^d,2^
OCN	39 ± 6ª^,1^	28 ± 5^b,2^	30 ± 9^b,1^	–	–
30 days					
Capsule thickness	152 ± 5^b,2^	203 ± 26^b,3^	201 ± 30^b,3^	274 ± 23^a,3^	160 ± 17^b,2^
ICs	241 ± 42^b,3^	278 ± 26^b,2^	272 ± 46^b,2^	584 ± 31ª^,3^	196 ± 27^b,2^
IL-6	100 ± 9^b,3^	119 ± 14^b,3^	119 ± 12^b,3^	219 ± 21ª^,3^	89 ± 17^b,2^
OCN	50 ± 6^a,2^	39 ± 6^b,3^	39 ± 9^b,2^	–	–
60 days					
Capsule thickness	120 ± 11^b,2^	129 ± 5^b,4^	126 ± 8^b,4^	185 ± 9ª^,4^	94 ± 8^b,3^
ICs	160 ± 9^b,3^	152 ± 8^b,3^	148 ± 11^b,3^	287 ± 11ª^,4^	98 ± 11^b,3^
IL-6	67 ± 9^b,4^	70 ± 12^b,4^	80 ± 15^b,4^	157 ± 15ª^,4^	61 ± 8^b,3^
OCN	72 ± 6^a,3^	61 ± 7^b,4^	61 ± 7^b,3^	-	-

Comparison between groups in the same period is indicated by superscript letters on the line. Different letters represent statistically significant difference. Superscript numbers indicate the comparison between periods in the same group in the columns. Different numbers represent statistically significant difference.

Tukey’s test (*P* ≤ 0.05).

**Figure 2:**
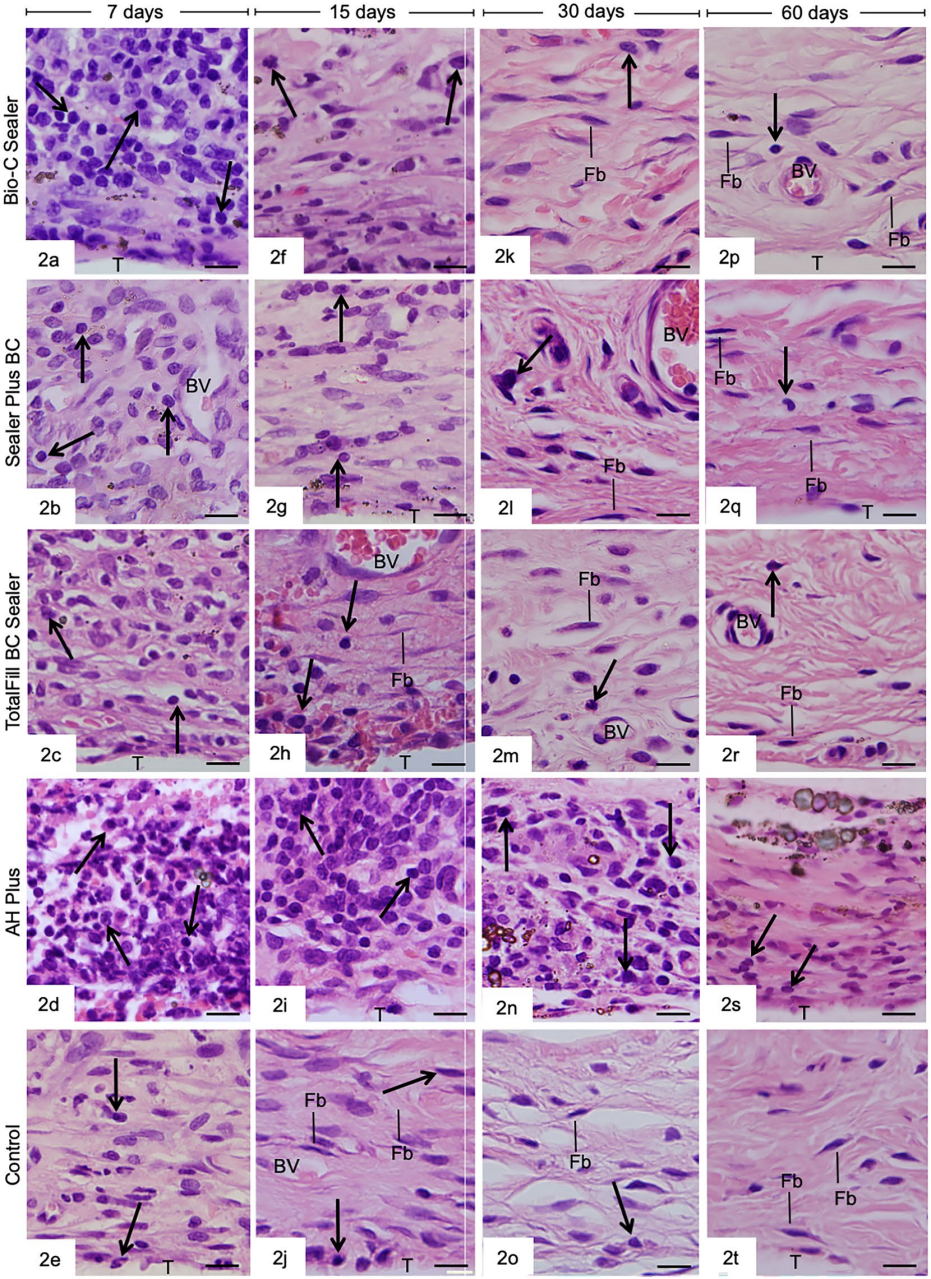
Photomicrographs of sections showing capsules adjacent to the implanted tubes (T) in the subcutaneous tissue, in high magnification, at 7 (Fig. 2a–e), 15 (Fig. 2f.–j), 30 (2 k–o), and 60 (2p–t) days. Figures 2a–e: the capsules show numerous inflammatory cells (arrows), BV, blood vessels; Figs. 2p–t: the capsules have typical fibroblasts (Fb) between bundles of collagen fibbers (CF); few inflammatory cells (arrows) are seen in the capsules. HE. Bars = 13 µm.

The sections showing inflammatory reactions caused by sealers in the connective tissue are presented in Fig. 2. At 7 days, the examination at high magnification revealed several mononuclear inflammatory cells, particularly macrophages, lymphocytes, and plasma cells in the thick capsules around the specimens (Fig. 2a–e). In addition, an enhanced density of inflammatory cells was observed in the capsules of AHP specimens (Fig. 2d). At 15 days, some fibroblasts were seen among inflammatory cells in the capsules of all groups, except in AHP specimens. In addition, the capsules contained numerous inflammatory cells (Fig. 2f–j). After 30 (Fig. 2k–o) and 60 (Fig. 2p–t) days, the capsules around SP, BIOC, TF, and CG specimens contained predominantly fibroblasts dispersed between bundles of collagen fibbers and a few inflammatory cells. In contrast, in AHP specimens, several inflammatory cells were still observed (Fig. 2n,s).

The analysis of the numerical density of inflammatory cells (ICs) (Table 2) showed that the highest IC were observed in the AHP specimens (*P* < 0.05) in all periods. At 7 and 15 days, the IC number was significantly greater in the BIOC than in SP and TF specimens (*P* < 0.05), while no significant difference was observed between SP and TF groups in all periods (*P* > 0.05). At 30 days, there was no difference between the groups regarding the numerical density of inflammatory cells (*P* > 0.05), except for AHP that presented the highest values (*P* < 0.05). However, AHP showed gradual reduction in the numerical density of ICs over time (*P* < 0.05). BIOC showed a significant reduction in the number of ICs from 15 days when compared to 7 days, while SP, TF, and CG showed statistical differences from 30 days (*P* < 0.05).

### Immunohistochemical detection, von Kossa reaction and analysis under polarized illumination

**Figure 3:**
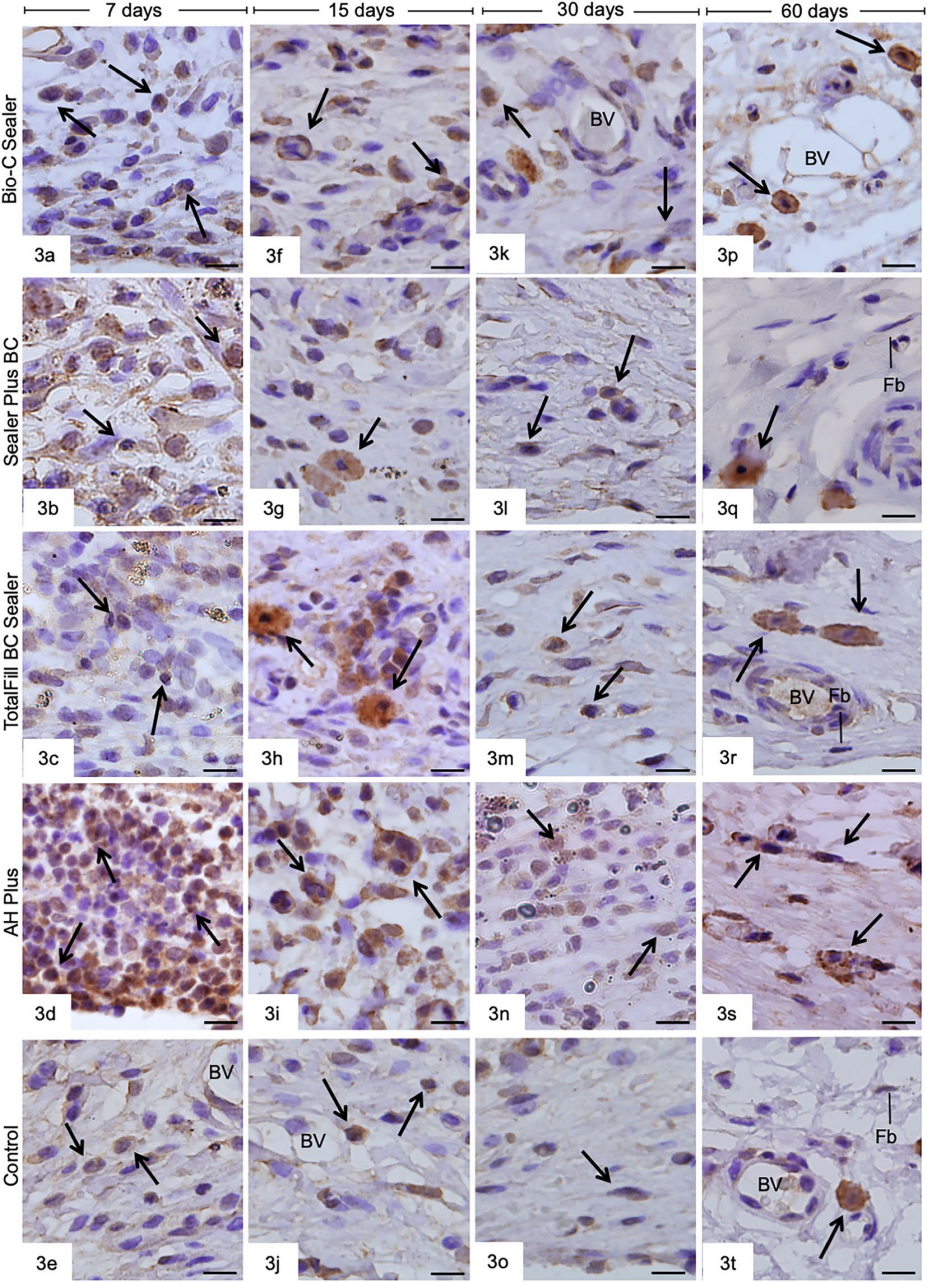
Photomicrographs showing capsules adjacent to the tubes implanted in the subcutaneous tissue for 7 (Fig. 3a–e), 15 (3f–j), 30 (3 k–o), and 60 (Figs. 3p–t) days. The sections were subjected to immunohistochemistry to detect IL-6-imunopositve cells (in brown) and hematoxylin. BV, blood vessels. Bars = 13 µm.

Figure 3 presents the sections subjected to the immunohistochemistry for detecting IL-6. The sections are presented in Fig. 3. In addition, several immunolabeling inflammatory cells were present in the capsules at 7 and 15 days (Fig. 3a–j), while few immunostained fibroblasts and inflammatory cells were seen after 30 and 60 days (Fig. 3k–t).

According to Table 2, the number of IL-6-immunolabeling cells was significantly higher in the AHP than in other groups in all periods (*P* < 0.05). At 7 and 15 days, the immunolabelling for IL-6 was significantly higher in the BIOC than SP, TF and CG (*P* < 0.05). SP and TF showed similar immunolabelling of IL-6 (*P* > 0.05) in all periods, showing greater values than CG at 7 and 15 days (*P* < 0.05). At 30 and 60 days, SP, TF, BIOC and CG were similar (*P* > 0.05). From 7 to 60 days, a significant decrease in the immunolabelling of IL-6 was observed in the capsules adjacent to dentin tubes filled with the sealers (*P* < 0.05). There were no significant differences in the number of immunolabeling cells between the periods of 15 and 30 days for the CG (*P* > 0.05).

**Figure 4:**
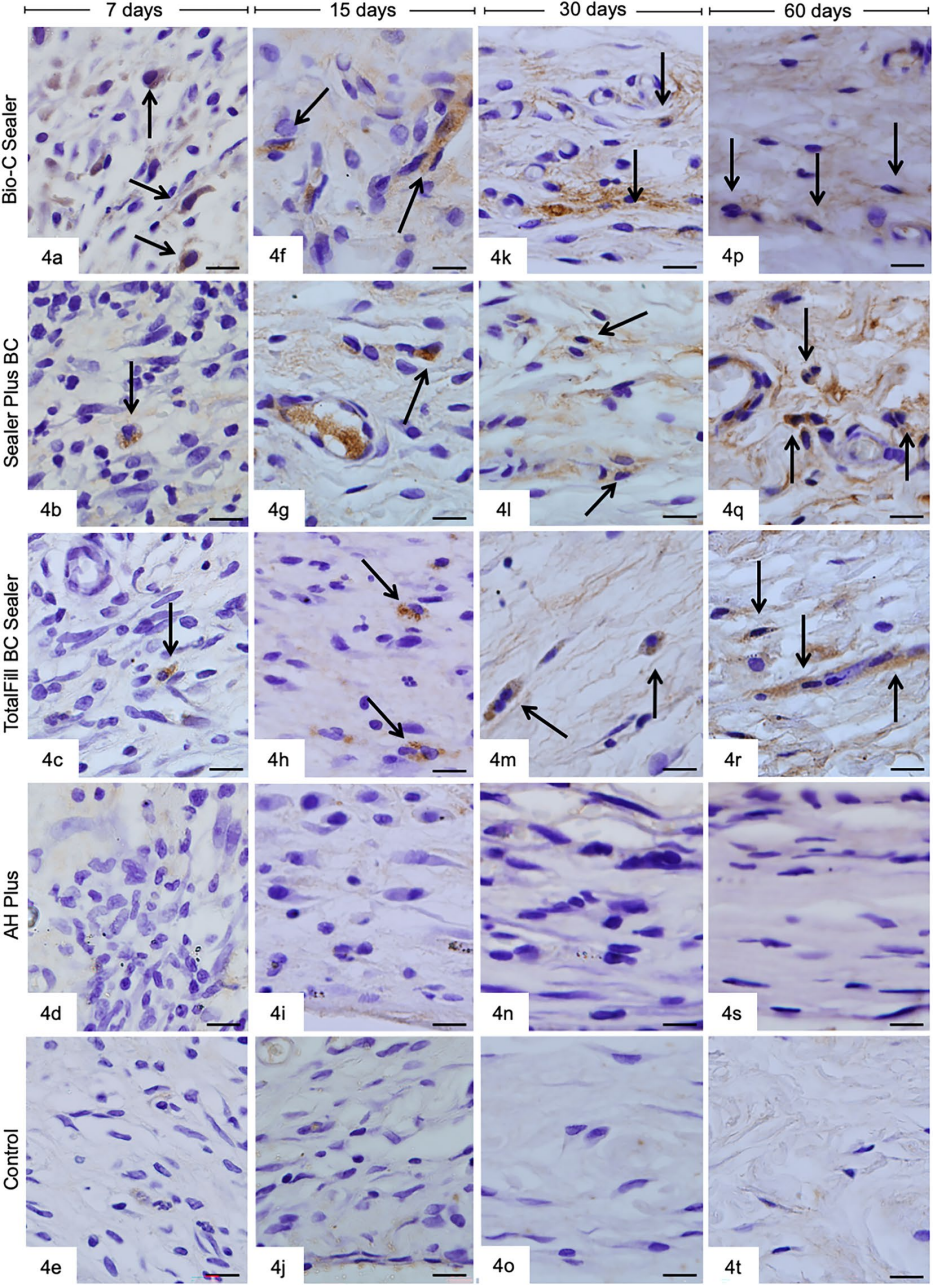
Sections showing capsules adjacent to the tubes implanted for 7 (Fig. 4a–e), 15 (4f.–j),30 (4 k–o), and 60 (Fig. 4p–t) days. The sections were subjected to immunohistochemistry for osteocalcin (brown-yellow color) and hematoxylin. Bio-C Sealer, Sealer Plus BC, TotalFill BC Sealer contain immunolabeling fusiform/elliptical cells (arrows). No immunolabeling cells are observed in AH Plus and control groups (Fig. 4d–s,e–t). Bars = 13 µm.

The sections subjected to immunohistochemistry to detect OCN-immunolabeling cells (brown-yellow) are presented in Fig. 4. Few OCN-immunolabeling cells were observed in the capsules of BIOC, SP, and TF groups at all time points. In contrast, immunolabeling cells for OCN were not seen in the capsules of AHP and CG groups in the different periods (Fig. 4a–t). At 30 and 60 days, immunolabeling was also observed in the extracellular matrix (Fig. 4k–m,p,r).

According to Table 2, the BIOC group exhibited the highest values of OCN-immunolabeling cells in the capsules in all periods (*P* < 0.05). At 7 days, the immunolabelling of OCN was significantly greater in the TF group than in SP specimens (*P* < 0.05). However, there was no significant difference between SP and TF at 15, 30 and 60 days (*P* > 0.05). From 7 to 60 days, a significant increase in the number of OCN-immunolabeling cells was observed in the capsules of SP samples (*P* < 0.05). BIOC and TF showed similar values between 7 and 15 days (*P* > 0.05), while a significant increase in immunolabeling occurred at 30 and 60 days (*P* < 0.05). At all-time points, OCN-immunolabeling cells were not observed in the capsules of AHP and CG groups.

**Figure 5:**
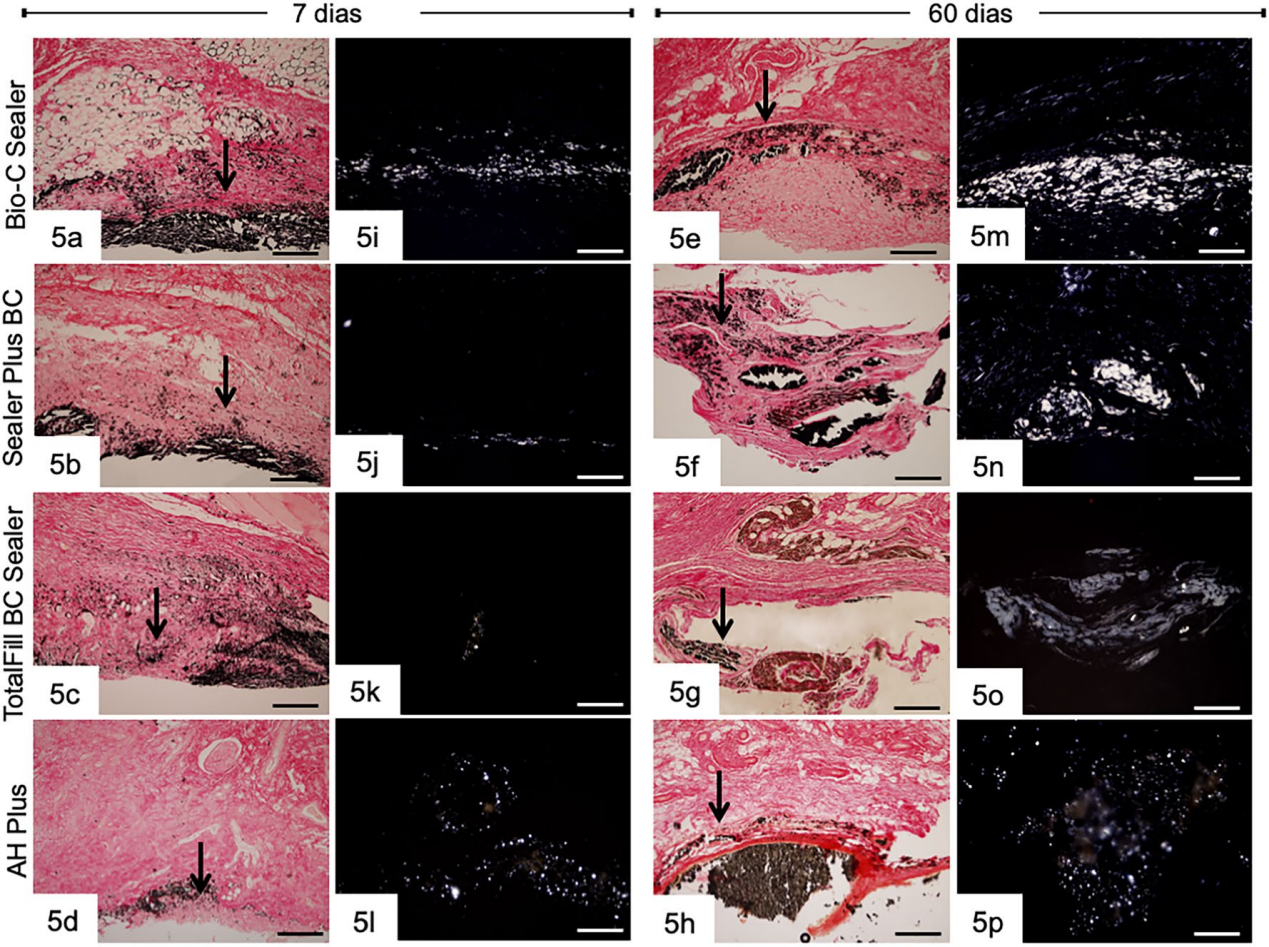
Sections submitted to von Kossa and picrosirius red. Bio-C Sealer, Sealer Plus BC, TotalFill BC Sealer and AH Plus exhibit positive structures to von Kossa method (black/brown color), at 7(Fig. 5a–e) and 60 (Fig. 5e–h) days. Figures 5i–l and 5 m–p -unstained sections analyzed under polarization illumination. Birefringent deposits are present in the capsules adjacent to the materials. Bars = 285 µm.

The sections with picrosirius red submitted to von Kossa method and sections analyzed by polarization microscope are presented in Fig. 5. The capsules of BIOC, SP, TF, and AHP exhibited von Kossa-positive structures at 7 (Fig. 5a–d) and 60 (Fig. 5e–h) days. Unstained sections analyzed under polarized light revealed birefringent structures in the capsules of BIOC, SP, TF, and AHP samples (Fig. 5i–l,m–p). Birefringent structures were not found in the capsules of CG.

### Porosity and dentin-material interface analyses

**Figure 6:**
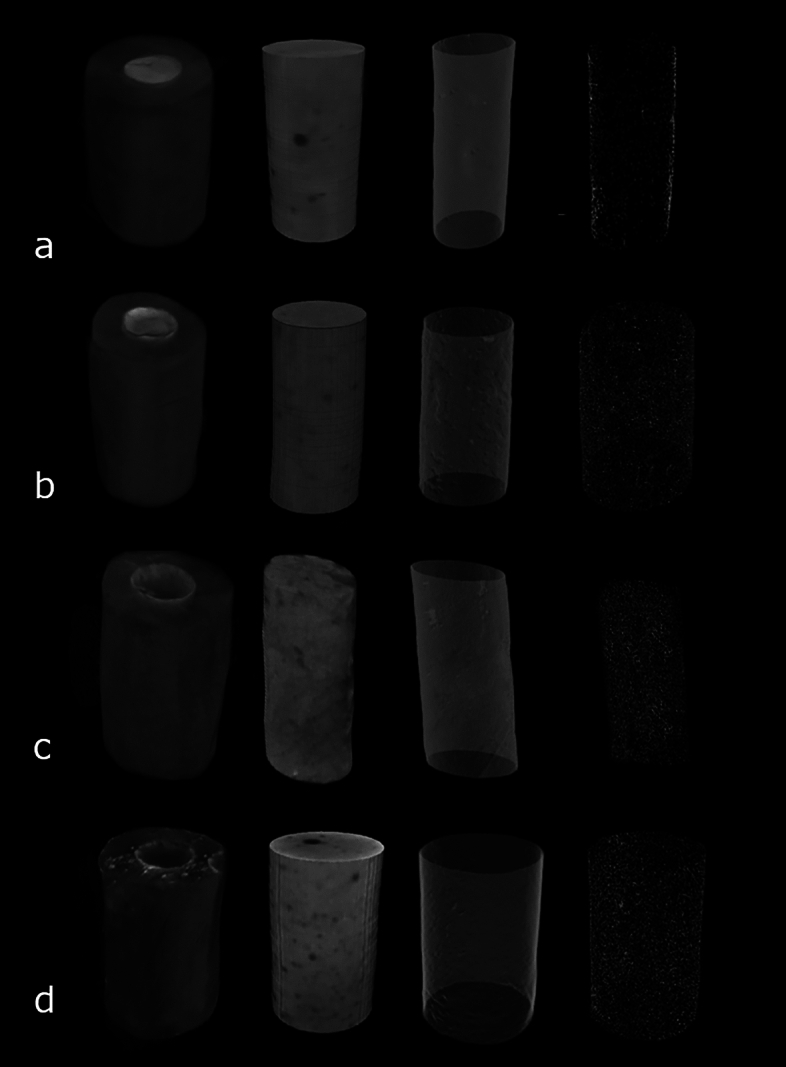
3D models representing the porosity of the dentin tubes filled with the sealers: (**a**) Bio-C Sealer, (**b**) Sealer Plus BC, (**c**) TotalFill BC Sealer and (**d**) AH Plus.

Porosity values observed in micro-CT are shown in Table 3. Porosity increased at 60 days compared with the baseline for all groups (*P* < 0.05). For BIOC, porosity in the baseline was similar to 7 days (*P* > 0.05), and lower than 15 days (*P* < 0.05), while an increase on the porosity was shown at 30 and 60 days (*P* < 0.05). For SP, the porosity values were similar in the baseline and at 7 days (*P* > 0.05), which showed lower values (*P* < 0.05) than at 15, 30 and 60 days (*P* > 0.05). TF had low values of porosity in the baseline, similar to 7 days (*P* > 0.05), and lower than at 15, 30 and 60 days (*P* < 0.05). AHP had the lowest values of porosity in the baseline (*P* < 0.05), and no significant differences were observed among the other time points (*P* > 0.05). When comparing the sealers, their porosity was similar in the baseline (*P* > 0.05), while AHP had the highest and SP the lowest values at 7 days (*P* < 0.05). At 15 and 30 days, AHP had more porosity than TF, while at 60 days, BIOC had the highest porosity (*P* < 0.05). The 3D models created in the CTVox program with the different sealers are presented in Fig. 6.

**Table 3:** Mean and standard deviation of porosity and interface (voids) in the different time intervals observed in the sealers.

	BIOC	SP	TF	AHP
Porosity (%)				
Baseline	0.71 ± 0.27^4^	0.81 ± 00.9^2^	0.66 ± 0.19^4^	0.84 ± 0.18^2^
7 days	1.34 ± 0.43^b,3,4^	0.52 ± 0.42^c,2^	1.11 ± 0.25^b,3,4^	2.02 ± 0.28^a,1^
15 days	2.14 ± 0.47^ab,3^	2.25 ± 0.95^ab,1^	1.45 ± 0.38^b,2,3^	2.85 ± 1.12^a,1^
30 days	3.40 ± 0.59^a,2^	2.45 ± 0.42^ab,1^	1.83 ± 0.69^b,2^	2.68 ± 0.78^a,1^
60 days	4.66 ± 0.68^a,1^	2.87 ± 0.17^b,1^	3.07 ± 0.7^b,1^	1.91 ± 0.61^c,1^
Interface (%)				
Baseline	1.01 ± 0.30^3^	1.43 ± 0.3^3^	1.80 ± 1.95^2^	0.81 ± 0.42^3^
7 days	1.20 ± 1.17^3^	0.99 ± 0.26^3^	1.53 ± 0.63^2^	0.95 ± 0.49^3^
15 days	2.52 ± 1.62^2,3^	1.20 ± 0.39^3^	2.82 ± 1.46^2^	1.69 ± 0.78^3^
30 days	3.60 ± 0.83^2^	3.26 ± 0.21^2^	2.78 ± 0.96^2^	2.95 ± 0.19^2^
60 days	5.60 ± 0.41^1^	5.69 ± 0.43^1^	5.75 ± 0.37^1^	5.39 ± 055^1^

The presence of different superscript letters on the same line represents statistically significant difference between groups in the same period. Superscript numbers indicate the comparison between periods in the same group in the columns. Different numbers represent statistically significant difference.

Tukey’s test (*P* ≤ 0.05). Baseline is represented by sealers after complete setting (37 ± 1 °C, 95 ± 5% relative humidity).

**Figure 7:**
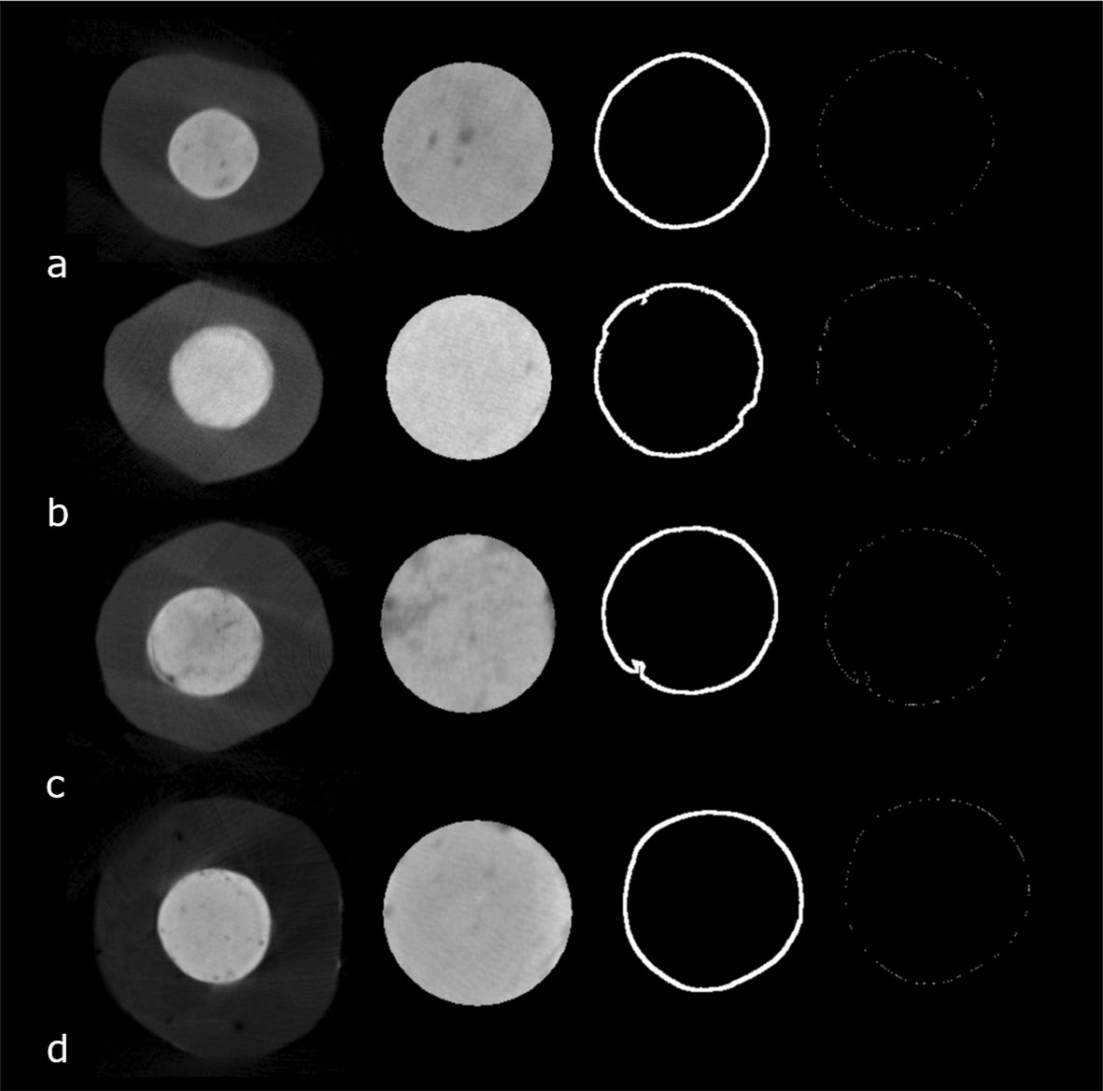
Microtomographic images representing the presence of voids and gaps in the interface dentin/sealer of (**a**) Bio-C Sealer, (**b**) Sealer Plus BC, (**c**) TotalFill BC Sealer and (**d**) AH Plus.

Table 3 also shows the interface evaluation of the sealers. All groups showed higher values in the percentage of voids at 60 days when compared to the baseline (*P* < 0.05). For BIOC and SP, the lowest values of interface voids were observed in the baseline, which was similar to the periods of 7 and 15 days (*P* > 0.05), while an increase in the values was observed at 30 and 60 days (*P* < 0.05). TF had the greatest values of interface voids at 60 days (*P* < 0.05), while the other time points were similar among them (*P* > 0.05). For AHP, the highest values were observed at 60 days (*P* < 0.05), followed by 30 days (*P* < 0.05), while the baseline, 7, and 15 days had low similar values (*P* > 0.05). Regarding the comparison among groups, BIOC, SP, TF, and AHP exhibited a similar percentage of voids during all time (*P* > 0.05). Images captured from CTAn software are presented in Fig. 7.

## Discussion

The present study evaluated biocompatibility and bioactivity of root canal sealers using dentin tubes to enable interactions between ready-to-use bioceramic sealers and dentin interface, simulating clinical application, as previous studies^12,15,19^. Although a previous study applied a similar methodology to evaluate reparative cements^19^, no other study evaluated biocompatibility and bioactivity of ready-to-use bioceramic sealers with a dentin tube model, besides their porosity and interface in different periods using micro-CT. The null hypothesis was rejected since differences were observed in the biological and physicochemical properties of the sealers. Bioceramic sealers induced less inflammation, showed bioactive potential, and had differences regarding their porosity when compared to AHP; however, there was no difference between them for the interface analysis.

At 7 days, all sealers presented a thick capsule exhibiting a high density of inflammatory cells around the dentin tubes and enhanced IL-6 immunolabeling. The initial reaction observed in the CG (empty tubes) has been associated with surgical trauma^11,24,25^. The alkaline pH of bioceramic sealers and the formation of calcium hydroxide can promote the recruitment of inflammatory cells and the production of cytokines^11,26^. Thus, the alkaline pH and calcium hydroxide formation may be related to the greater inflammatory infiltrate and IL-6 immunolabeling in the capsules around the tubes filled with sealers than CG specimens. However, this alkaline environment provided by bioceramic sealers can positively affect apical healing, contributing to the formation of mineralized tissues^27^.

BIOC had a thickness capsule with a higher value of inflammatory cells and IL-6-immunolabeling cells than SP and TF on the 7th and 15th day. There was a decrease in the number of ICs and the IL-6 immunolabeling on 30th day, showing an intense remodeling in the capsules of all groups, except AHP in the initial period. This response could be explained by the high solubility and flow of BIOC^7^, indicating that substances released by this sealer may be responsible for tissue damage caused initially. Furthermore, BIOC presents polyethylene glycol as dispersant agent^3^, which can also interfere with its biological properties.

SP and TF showed no statistical difference in capsule thickness, number of ICs and immunolabeling in all periods, demonstrating proper biological properties. After 60 days, the bioceramic sealers had no statistical difference between them and the CG. A significant decrease in capsule thickness was detected in all groups over time. However, only the bioceramic sealers were involved by thin capsules, exhibiting a thickness of less than 150 µm. These findings indicate that BIOC, SP and TF are biocompatible and have a lower inflammatory reaction when compared to AHP, according to studies using polyethylene tubes in the subcutaneous tissue of rats^3,4^.

IL-6 is a cytokine produced by fibroblasts, macrophages, and neutrophils in response to aggressions and infections^11,25^. There was a significant gradual decrease in the numerical density of ICs and the number of IL-6-immunolabeling cells in the capsules with concomitant formation of dense connective tissue containing collagen fibber bundles in the bioceramic sealers. IL-6 plays an essential role in the inflammatory response^11,26^. Other studies have associated the IL-6 with the intensity of the inflammatory reaction caused by calcium silicate sealers implanted in subcutaneous tissues^3,4,28,29^.

AHP, an epoxy resin-based root canal sealer, was used in this study as a reference material due to its physicochemical properties^30^. AHP presented the highest number of IL‐6‐immunolabeling cells and inflammatory cells in all periods. The number of these cells reduced over time. The mechanism of this inflammatory reaction can be related to AHP composition that delays the healing process by the epoxy resin released^31^ and the amines’ presence to accelerate the polymerization^32^. Thus, there is evidence that bioceramic sealers exhibit lower cytotoxicity^2^ and better biocompatibility than AHP sealer^3,31^.

The bioactive potential was assessed by the von Kossa histochemical method associated to the immunohistochemical reaction of osteocalcin, since only the von Kossa method alone is not proper to identify and quantify bonelike minerals^33^. Osteocalcin is utilized as a marker for mature osteoblasts and can bind to calcium, which plays a role in the mineralization process^34^.

In all periods the bioceramic sealers had immunolabeling for osteocalcin and presented structures positive to von Kossa staining with calcium deposits in the adjacent capsules. AHP exhibited von Kossa positivity due to its calcium release^3,35^. However, AHP did not show positive marking for osteocalcin at any period, suggesting no bioactivity, in agreement with a previous study^3^. Therefore, bioceramics may stimulate mesenchymal cells in subcutaneous tissue to express the osteoblast-like phenotype. The lack of OCN-positive immunostaining in the capsules formed for both AHP and control groups supports this hypothesis. Additional analysis by scanning electron microscope (SEM) of dentin tubes implanted in the subcutaneous tissue of rats can confirm the bioactive potential in vivo, verifying the formation of a mineral interfacial layer between the cement and the dentin wall of the tube^12,36^.

Bioceramic materials can release calcium and hydroxyl ions, contributing to the tissue repair and mineralization process^37^. The reaction of calcium ions and carbon dioxide leads to calcite crystals, which are birefringent, justifying the findings that bioceramic sealers presented birefringent structures in all periods. BIOC, SP and TF showed biocompatibility due to the reduction of the inflammatory process over time and the increase in collagen. In addition, all materials were bioactive showing osteocalcin-immunolabeling cells and von Kossa stain-positive structures in the capsules.

In silico toxicity and immunological interactions of components of calcium silicate-based and epoxy resin-based endodontic sealers was performed including IL-1β, IL-6, IL-8, IL-10 and TNF-α. The predictions and molecular docking pointed the higher toxicity and greater interaction with mediators of periapical inflammation of the main test compounds from AHP compared to those from calcium silicate-based sealer^38^. In addition, the evaluation of bioactivity, cytocompatibility, and anti-inflammatory potential showed expression levels of proinflammatory cytokines IL-6 and IL-8 higher in AHP than in bioceramic sealers favoring the calcified nodule formation from human periodontal ligament stem cells^39^. These findings corroborate our study.

Root canal sealers with low porosity besides low voids or gaps in the interface between the sealer and the dentin are expected to provide an adequate sealing in order to avoid microbial and fluid leakage^40,41^. All sealers had porosity and interface voids values below 6%, which may be correlated with the high viscosity and small particle size of these sealers^23^. Our findings corroborate a previous study, which observed low and similar voids for AHP and bioceramic sealers^6^.

The present study used an in vivo method in rats, in addition to three-dimensional evaluations, enabling the assessment of biological and volumetric properties of ready-to-use bioceramic sealers. Experimental models using rats are important since the basic immunobiology of these animals is similar to humans^42^, while three-dimensional analysis allows an evaluation of important properties for the choice of endodontic material in its clinical application^43^. However, some limitations must be considered, since the histological reactions in the subcutaneous connective tissue present differences in relation to the reactions in the pulpal and periapical tissues^38^. Therefore, clinical studies are important to analyze the behavior of these materials in their clinical applications.

## Methods

### Approval by the research ethics committee

The following methods were carried out in accordance with the Declaration of Helsinki and this study was approved by the Research Ethics Committee of the University (protocol number: 12647319.8.0000.5416). Informed consent was waived by Research Ethics Committee of the University as all teeth used in this study were obtained from the Human Teeth Bank of the University. In addition, the Animal Experimentation Ethics Committee approved this protocol in the University (#04/2019). The experiment and analysis methods were conducted in accordance with ARRIVE guidelines 2.0 (Animal Research: Reporting of in vivo Experiments). All animal experiments followed all relevant guidelines and regulations.

### Preparation of dentin tubes

This study used single-root human teeth to produce dentin tubes. All the tubes were passed through the standardization process, and the samples that were out of pattern were rejected. The dentin tubes were sectioned with a precision cutting machine (ISOMET; Buehler, Lake Bluff, IL, USA) and prepared using Gates-Glidden burs #5 (Dentsply Sirona, Charlotte, NC, USA). Specimens with 5 mm in length, 1.3 mm in internal diameter, and 1.5 mm wall thickness were obtained. The measurements were confirmed with the aid of a digimatic caliper (Mitutoyo Corporation, São Paulo, SP, Brazil) and an iwanson caliper (Golgran Millennium, São Caetano do Sul, SP, Brazil). The dentin tubes were submitted to a protocol to remove the “smear layer”, using 17% EDTA, sodium hypochlorite 1%, and distilled water, and then were sterilized in the autoclave (Cristófoli Equipamentos de Biossegurança, Campo Mourão, PR, Brazil) using a test tube with 100 mL distilled water, wrapped in surgical grade paper (15 minutes, 1700 W, 127 °C), with no drying cycle. After autoclaving, the tubes were stored in an oven (37 °C, 95% humidity) and kept hydrated with distilled water until the filling with the sealers.

### Biological properties analysis

#### Experiment design

Thirty-two adults male Holtzman rats (Rattus norvegicus albinus) weighing between 270 and 300g were used and housed in polyethylene cages, and maintained under a 12:12 light-dark cycle at controlled temperature (23±2 °C) and humidity (55±10%), with food and water provided ad libitum. The animals were distributed in five groups (n = 6 tubes per group): dentin tubes were filled with the endodontic sealers (Table 1) and empty tubes were used as a control (control group - CG). The sample size was calculated using G*Power 3.1.7 software (Heinrich-Heine Universität, Düsseldorf, Germany). The calculation was based on an alpha-type error of 0.05 and a beta power of 0.99 for all variables. Previous studies were employed to determine the specific effect size of capsule thickness, 1.06^19^; number of inflammatory cells, 3.61^19^; interleukin-6-immunolabeled cells, 1.17^29^; and osteocalcin-immunolabeled cells, 2.04^44^. Six specimens per group/period were indicated as the ideal size required to observe significant differences.

Intraperitoneal anesthesia with xylazine hydrochloride (4 mg kg-1 body weight; União Química, São Paulo, SP, Brazil) and ketamine hydrochloride (80 mg kg-1 body weight, Virbac do Brasil, São Paulo, SP, Brazil) was applied in the animals. A 2 cm craniocaudal incision and tissue divulsion were performed in the dorsal region. Four tubes were inserted per animal corresponding to the different experimental groups^3,4^. The suture was performed with 4/0 silk thread (ETHICON, São José dos Campos, SP, Brazil). After 7, 15, 30, and 60 days post-implantation, the animals were euthanized with an overdose of anesthesia, and the implanted tubes with adjacent tissues were extracted. The dentin tubes were isolated from surrounding tissues and processed for micro-CT analysis to assess porosity and the dentin-sealer interface. Surrounding tissues were utilized for biocompatibility and bioactivity evaluations.

#### Histological procedures

The adjacent tissues were removed and immersed in a 4% formaldehyde solution, buffered with 0.1 M sodium phosphate and pH 7.2 for 72 hours. After fixation, the specimens were dehydrated, diaphanized, immersed in liquid paraffin (60 °C) for 4 hours, and embedded in paraffin. Longitudinal sections with a thickness of 6 µm were obtained. Non-serial sections were stained with hematoxylin-eosin (H&E) for estimated the number of inflammatory cells and capsules’ thickness. Additional non-serial sections were mounted on slides treated with 4% silane (Sigma-Aldrich, Saint Louis, MO, USA) and underwent immunohistochemistry to detect osteocalcin (OCN) and interleukin-6 (IL -6).

#### Numerical density of inflammatory cells

The number of inflammatory cells was obtained from three HE-stained sections (with a minimal distance of 100 µm between the sections) were captured under ×695 magnification (each field with 0.09 mm^2^). The number of inflammatory cells was computed using an image analysis program (Image-Pro Express 6.0 program; Olympus Corporation, Tokyo, Japan) and divided by total area (number of inflammatory cells per mm^2^)^3,4,11,28^.

#### Thickness of capsules

Three images of non-serial H&E-stained sections per specimen were captured at 65× magnification using a camera (DP- 71; Olympus Corporation, Tokyo, Japan) attached to a light microscope (BX-51; Olympus Corporation, Tokyo, Japan). Capsule measurements were conducted using an image analysis software (Image-Pro Express 6.0 program, Olympus Corporation, Tokyo, Japan) according to previous studies^3,4,24,28,29^.

#### Immunohistochemical detection of IL-6 and osteocalcin (OCN)

Deparaffinized sections were immersed in a 0.001 M sodium citrate buffer pH 6.0 and heated at 98 °C using a microwave for 30 minutes for detection of IL-6 and 10 minutes for detection of OCN. After cooling, the slides were washed with Tris-HCl 0.05 M buffer at pH 7.4, and the endogenous peroxidase was inactivated by treatment with 5% aqueous hydrogen peroxide solution for 20 minutes. Next, the sections were washed and incubated with 2% bovine serum albumin (BSA; Sigma-Aldrich, St Louis, MO, USA) for 30 minutes. Then, the sections were incubated overnight in a humid chamber at 4 °C with a primary mouse anti-IL-6 antibody (Abcam, Cambridge, UK, England, code Ab 9324) at 1:100 dilution or primary rabbit anti-osteocalcin antibody at 1:500 dilution (code SAB1306277; Sigma-Aldrich, St Louis, MO, USA). After washing, the sections were incubated with biotinylated anti-mouse IgG secondary antibody (LSAB, Dako., Carpinteria, CA, USA) at room temperature. The 3,3′-diaminobenzidine chromogen (ImmPACTTM DAB; Vector, Burlingame, CA, USA) revealed peroxidase activity, and then the sections were counterstained with hematoxylin Carazzi. The sections were incubated with non-immune serum instead of the anti-IL-6 and anti-OCN antibody as a negative control.

The number of IL-6 and OCN-immunolabeling cells was estimated in all specimens. In each specimen, a standardized field was captured at 695× magnification (0.09 mm^2^) using a digital camera attached (DP-71, Olympus Corporation, Tokyo, Japan) to the light microscope (BX-51, Olympus Corporation). In these images the number of immunolabeling cells (in brown/yellow color) per mm^2^ was calculated^3,4,28,29^.

#### von Kossa reaction and analysis under polarized light

The von Kossa method was used for calcium deposit detection in the capsules. The sections were dewaxed, hydrated, and immersed in 5% silver nitrate solution for 1 hour under sunlight. Then the sections were rinsed in distilled water for 3 minutes, immersed in 5% sodium hyposulfite solution for 5 minutes. After washing with distilled water for 5 minutes, the sections were stained with picrosirius-red for 1 hour, dehydrated, and mounted in a resinous medium^3,45^.

The evaluation of the birefringent structures in the capsules was performed using unstained sections examined under polarized light (BX51; Olympus Corporation, Tokyo, Japan)^3,4,28,29^.

#### Physicochemical properties analysis

After the periods of implantation of 7, 15, 30 and 60 days, the dentin tubes filled with SP, BIOC, TF, and AHP (n = 6 per group) were extracted from the subcutaneous tissue of rats. The sample size was calculated using G*Power 3.1.7 software (Heinrich-Heine Universität, Düsseldorf, Germany). One-way ANOVA was employed with an alpha-type error of 0.05, and a beta power of 0.99 for all variables. A previous study^19^ was employed to calculate the specific effect size for each variable: 2.21 for porosity and 1.11 for dentin-material interface. In order to observe significant difference between the experimental groups and CG, six specimens per group/period were indicated as the ideal number required. The dentin tubes were kept for 24 hours with gauze moistened in distilled water and stored in an oven (37 °C, 95% humidity). For comparison, samples (n = 6) were prepared using new dentin tubes filled with freshly sealers (baseline), which were kept for 48 hours with gauze moistened in distilled water and stored in an oven (37 °C, 95% humidity) for a complete set^19^.

The specimens were evaluated using microcomputed tomography (micro-CT, SkyScan 1176; Bruker-microCT, Kontich, Belgium), with the following parameters: 313 µA current, 80 kV, pixel size 9 µm, and 360° rotation with a Cu + Al filter. Image reconstruction was carried out using NRecon program (V1.6.4,7; Bruker-MicroCT, Kontich, Belgium) with correction parameters for beam hardening, smoothing and ring artifacts set for each material. Image analysis was performed using CTAn software (V1.11.8; Bruker-MicroCT, Kontich, Belgium).

#### Porosity

The porosity of the sealers was measured in cubic millimeters and as a percentage. The values of porosity for each sealer and time period were compared to the baseline. CTAn and CTVol software (V2.0; Bruker-MicroCT, Kontich, Belgium) were used to create 3D models of the filled cavities^19,21^.

#### Dentin-material interface

The method for evaluating the differences in void percentages at the interface between the dentin surface of the root canal walls and the sealers was based on previous studies^6,19^. The 3D distribution of interface voids within a predefined volume of interest (VOI), including the canal wall dentin and the sealer was calculated for each group and compared to the baseline. Voids starting from a size of 9 μm within the VOI were detected using the threshold grey level. 3D models of the voids were then created using CTAn software.

### Statistical analysis

All data were statistically analyzed using GraphPad Prism 9 software (Jandel Scientific, Sausalito, CA, USA). The data passed the Kolmogorov-Smirnov normality test. Biological property data were analyzed with two-way ANOVA followed by Tukey’s test, while porosity and interface analysis data were subjected to one-way ANOVA with Tukey’s test. A significance level of *P* ≤ 0.05 was accepted.

## Data Availability

The datasets of the present research are available and can be requested from the corresponding author.
